# Progestin vs. Gonadotropin-Releasing Hormone Antagonist for the Prevention of Premature Luteinizing Hormone Surges in Poor Responders Undergoing *in vitro* Fertilization Treatment: A Randomized Controlled Trial

**DOI:** 10.3389/fendo.2019.00796

**Published:** 2019-11-22

**Authors:** Qiuju Chen, Weiran Chai, Yun Wang, Renfei Cai, Shaozhen Zhang, Xuefeng Lu, Xiaojing Zeng, Lihua Sun, Yanping Kuang

**Affiliations:** ^1^Department of Assisted Reproduction, Shanghai Ninth People's Hospital, Shanghai Jiaotong University School of Medicine, Shanghai, China; ^2^Centre of Assisted Reproduction, Shanghai East Hospital, Tongji University, Shanghai, China

**Keywords:** premature LH surge, GnRH antagonist, progestin-primed ovarian stimulation, poor responders, controlled ovarian stimulation

## Abstract

**Objective:** Progestin was recently used as an alternative of gonadotropin-releasing hormone (GnRH) analog for preventing premature luteinizing hormone (LH) surge with the aid of vitrification techniques, however, limited data were available about the potential of progestin in poor responders undergoing *in vitro* fertilization (IVF)/intracytoplasmic sperm injection (ICSI) treatment. We performed a randomized parallel controlled trial to investigate the difference of progestin and GnRH antagonist in poor responders.

**Methods:** A total of 340 poor responders who met with Bologna criteria were randomly allocated into the progestin-primed ovarian stimulation (PPOS) group and GnRH antagonist group. Fresh embryo transfer was preferred in the GnRH antagonist group and freeze-all was performed in the PPOS group. The primary outcome was the incidence of premature LH surge, secondary outcomes were the number of retrieved oocytes, the number of viable embryos and the pregnancy outcomes.

**Results:** The results showed that the incidence of premature LH surge in PPOS group was lower than that in antagonist group (0 vs. 5.88%, *P* < 0.05). In PPOS group, the average numbers of oocytes and viable embryos were comparable to those in GnRH antagonist group (3.7 ± 2.6 vs. 3.4 ± 2.4; 1.6 ± 1.7 vs. 1.4 ± 1.3, *P* > 0.05), the live birth rate was similar between the two groups (21.8 vs. 18.2%, RR 1.25 (95% confidence interval 0.73, 2.13), *P* > 0.05).

**Conclusions:** The study demonstrated that PPOS had a more robust control for preventing premature LH rise than GnRH antagonist in poor responders, but PPOS in combination with freeze-all did not significantly increase the probability of pregnancy than GnRH antagonist protocol for poor responders.

## Introduction

Since the beginning of ovarian stimulation for *in vitro* fertilization (IVF), the management of poor ovarian response has been a baffling riddle for clinicians (1). The overall incidence of poor response is reported 15–16% among all stimulated IVF/intracytoplasmic sperm injection (ICSI) cycles in U.S clinics (2). The wide-used criteria for poor responders are the Bologna criteria, which define the specified population with risks of expected poor response (advanced age, diminished ovarian reserve) and previous poor response (3). Poor response is always associated with poor treatment outcomes from both natural cycle IVF and traditional stimulation cycles (4, 5).

How to control the premature luteinizing hormone (LH) surge and premature ovulation in poor responders has long being an issue in IVF treatment (6). Gonadotropin-releasing hormone (GnRH) antagonist has been used to suppress pituitary activity and prevent premature LH surges during controlled ovarian stimulation since 1990s (7, 8), and it is beneficial for the poor responders due to its advantage of less suppression in the early follicular phase (9–11). GnRH antagonist is reported about 0.34–8.0% failure to control premature LH surge in ovulatory women, the predominant risk factors of GnRH antagonist failure include the increased age, diminished ovarian reserve (DOR) and poor response to gonadotropin (12–16).

Although progestin has been widely applied to controlling ovulation in hormonal contraception for more than 50 years, it has been extended to use for preventing premature ovulation in IVF since 2013, with the aid of the progresses in vitrification techniques (17–22). Our recent researches demonstrated that the continuous administration of progestin showed a gradually decreased LH level during the ovarian stimulation, with a low incidence of LH surge and premature ovulation in ovulatory women (0.15%) and in poor responders (3.0%) (18, 22). This progestin-primed ovarian stimulation (PPOS) in combination with deferred embryo transfer showed a comparable pregnancy outcome compared to the conventional protocols (such as short protocol and GnRH antagonist protocol) in infertile women with normal ovarian reserve (18–22). However, limited data were available to compare the efficacy of progestin and GnRH antagonist in blocking premature LH surge and premature ovulation in poor responders, so we performed a prospective single-central randomized controlled trial (RCT) to investigate the potential of progestin and GnRH antagonist used for the poor responders undergoing IVF/ICSI treatment.

## Methods

We conducted a prospective RCT at the department of assisted reproduction of Shanghai Ninth People's Hospital affiliated Shanghai Jiaotong University School of Medicine from March 2017 to September 2018. The study had been approved by the Institutional Review Board (IRB) of Shanghai Ninth People's Hospital (Number: 2016-198-T142). The trial was registered in China Clinical Trial Registry on 18 March 2017 (Number: ChiCTR-IPR-17010906). Informed consents were obtained from each patient before any study procedure was performed, in accordance with good clinical practice. The study design, methods, inclusion and exclusion criteria have been described in detail elsewhere (23).

## Study Population

The study included the infertility women with age ≥22 and ≤42 years old, spontaneous menstrual cycle (21–35 days) and had at least one of the following indications for IVF or ICSI: tubal factor, male factor, diminished ovarian reserve, endometriosis or unexplained factors. The participants were diagnosed with poor responders according to the Bologna criteria (3), including at least two of three following criteria: (a) advanced women age (≥40 years) or any other risk factor for poor ovarian response (such as ovary surgery and ovarian endometrioma); (b) a previous poor response: with no more than three oocytes retrieved using the conventional stimulation protocols; (c) an abnormal ovarian reserve test results, including total antral follicle counts (AFC) in both ovaries <7 or serum anti-müllerian hormone (AMH) <1.1 ng/ml.

Exclusion criteria were the clinically significant systemic diseases such as renal failure and systemic lupus erythematosus, premature ovarian insufficiency, with known müllerian duct anomalies and with any contraindications to ovarian stimulation treatments. The women who had previous unsuccessful IVF attempts up to 5 times and were unable to comply with the study procedures were excluded.

## Randomization

Women who met the eligibility and completed a baseline visit were randomized into one of the two arms at a ratio of 1:1 during day 2–4 of their next menstrual cycle. Women were allowed to complete the baseline and randomization visit on the same day. The allocation sequences were generated by investigators utilizing computer-generated random number. Both investigators and participants were aware of the allocations during IVF treatment. The physicians and embryologists involved in the procedures of oocyte retrieval and embryo transfer were blinded to the treatment assignments throughout.

## Treatment

### GnRH Antagonist Protocol

Flexible GnRH antagonist protocol was performed as following: human menopausal gonadotropin (hMG) 150–225 IU daily was administered from menstrual cycle day 3, follicle monitor was performed 5 days later. When the dominant follicles reached the diameter about 14 mm, GnRH antagonist 0.125–0.25 mg daily was administered up to the trigger day. For the cases with low/normal body mass index (BMI) (<25.0 kg/m^2^) and low LH levels before GnRH antagonist administration (<2.0 mIU/ml), antagonist 0.125 mg daily was administered (15), and for the cases with higher BMI (≥25.0 kg/m^2^) or LH levels ≥2.0 mIU/ml, antagonist 0.25 mg was used daily up to the trigger day. The dose of hMG was adjusted according to the ovarian response. When the dominant follicles reached the diameter of 18 mm, the final stage of oocyte maturation was induced with triptorelin 100 μg S.C and human chorionic gonadotropin (hCG) 5,000 IU intramuscular injection. The oocyte retrieval was performed 36 h later.

### PPOS Protocol

hMG 150–225 IU and medroxyprogesterone acetate (MPA) 10 mg started daily from cycle day 3, follicles monitored 5 days later and the dose of hMG was adjusted according to the ovarian response. MPA dose was consistent up to the trigger day. When the dominant follicles reached the diameter of 18 mm, the final stage of oocyte maturation was induced with triptorelin 100 μg S.C and hCG 5,000 IU intramuscular injection. The oocyte retrieval was performed 36 h later.

### *In vitro* Fertilization and Embryo Culture

All follicles of more than 10 mm were retrieved, follicles were flushed three times at most if no cumulus oocyte complex presented. Standard insemination or ICSI was performed within 6 h of retrieval. Embryos were examined for the number and regularity of blastomeres and the degree of embryonic fragmentation on the third day. If available, one or two top-quality embryos (grade I/II 8-cell blastomere embryos) in GnRH antagonist group were transferred on the third day. The remaining top-quality embryos were frozen by vitrification, the non-top-quality embryos were extendedly cultured and only good morphology blastocysts were frozen on day 5 or 6. All top-quality cleavage embryos and the cryopreserved blastocysts were recorded as the viable embryos. In PPOS group, all top-quality embryos were frozen on the third day, the non-top-quality embryos were extendedly cultured and cryopreserved according to the same criteria as above.

### Endometrium Preparation and FET

The mild stimulation or hormone replacement therapy was used for endometrium preparation, no more than two embryos were transferred per cycle. For mild stimulation FET cycles, letrozole 2.5 mg was administered for 3 days, when the follicle matured and endometrial thickness was ≥8 mm, hCG 5,000 IU was administered and cleavage embryo transfer was arranged 4–5 days later. The blastocyst transfer was arranged 2 days later. Dydrogesterone 40 mg and micronized progesterone capsules 400 mg daily intravaginal were used for luteal support beginning on the third day after hCG injection (18).

For patients with thin endometrium or FET failures after stimulation cycles, hormone replacement therapy was recommended for endometrial preparation, ethinyl estradiol 75 mcg/day was orally administered from cycle day 3 onwards, when the endometrial lining was ≥8 mm thick, progesterone administration was started and cleavage embryo was transferred 3 days later. When pregnancy was achieved, the progesterone supplement continued until 10 weeks of gestation.

### Hormonal Measurement

Serum FSH, LH, estradiol and progesterone levels were monitored during the ovarian stimulation. Hormone levels were measured with chemiluminescence (Abbott Biologicals B.V., The Netherlands). Intra- and inter-assay coefficients of variation were 2.6 and 5.8% for FSH, 5.9 and 8.1% for LH, 6.3 and 6.4% for estradiol, and 7.9 and 10.0% for progesterone. The upper limit for estradiol measurement was set at no more than 5,000 pg/ml.

### Outcome Measurements

The primary outcome endpoint was the incidence of premature LH surge, defined as the serum LH >15 mIU/ml on the trigger day, with or without dominant follicle rupture and increased serum progesterone (23). Premature ovulation was defined as the dominant follicle rupture before the scheduled time. Elevated progesterone alone was not defined as the presentation of LH surge and was listed independently.

Secondary efficacy parameters included the number of oocytes and viable embryos, the clinical pregnancy rate, implantation rate and live birth rate. Clinical pregnancy was defined as the presence of intrauterine gestation sac at 7 weeks of gestation. Live birth was defined as the delivery of an infant after 28 weeks of gestation. The safety endpoints included the incidence of ovarian hyperstimulation syndrome (OHSS), miscarriage, ectopic pregnancy and pregnancy complications.

## Statistics

### Sample Size and Power Calculations

For the power calculation, previous studies reported that the incidence of premature LH surge in GnRH antagonist protocol was 8.0%, the recent data showed the incidence of premature LH surge and ovulation was 3.0% in poor responders using PPOS (22), therefore, we hypothesized that the administration of MPA would decrease the incidence of premature LH surges. The superiority margin was set as 4.0%. A sample size of 166 in each group would yield 90% power to establish superiority at the 0.01 level of significance, and 109 in each group yielded 90% power to establish superiority at 0.05 level of significance. Given the abundant clinical resources in our clinic, the number of participants was set as 170 in each group in this trial.

### Statistical Analysis

We utilized an intention-to-treat approach (ITT) to examine differences of the primary endpoint (the incidence of premature LH surge) and secondary endpoints. The difference between the incidence of premature LH surge for the two groups was tested via two-sided Chi-square test for independence. Parametric *t*-test or non-parament Mann-Whitney Wilcoxon methods were used as appropriate for continuous outcomes, and estimation of the median with 95% confidence interval (CI) was calculated. *P* < 0.05 is considered as the different significance. An exploratory analysis of transition probabilities for outcomes was also conducted to evaluate potential differences between treatment arms. Adverse events were monitored and recorded throughout the trial.

The full analysis and per protocol analysis were also executed in this trial and used as a complement.

## Results

### Participant Characteristics

**Figure 1:**
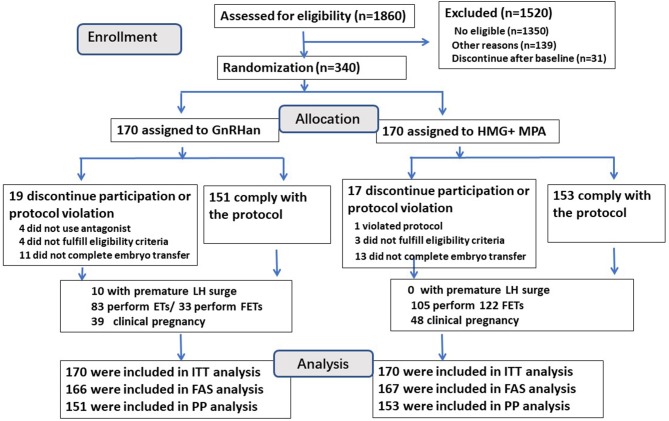
The flow chart of this trial.

A flowchart of the participant allocation is presented in Figure 1. A total of 1,860 women underwent screening, and 510 met the eligibility criteria. Of these women, 31 discontinued to treatment and 139 quitted for other reasons. A total of 340 women were randomly assigned to GnRH antagonist group or PPOS group, with 170 participants in each group.

Characteristics of participants, by treatment arm, are shown in Table 1. Treatment arms were similar with regard to the terms of basic characteristics (Age, BMI, AMH, infertility duration, basal FSH and AFC), the distribution of women with previous IVF attempts was slightly higher in the GnRH antagonist group but did not reach the significance (*P* > 0.05).

**Table 1:** The basic characteristics of women in the two groups.

	**GnRH antagonist (*n* = 170)**	**PPOS (*n* = 170)**
Age (years)	35.1 ± 4.1 (36.0–6.0)	34.8 ± 4.2 (35.0–6.0)
BMI (kg/m^2^)	21.7 ± 2.8 (21.2–4.0)	21.4 ± 2.7 (20.9–4.0)
Duration of infertility (years)	3.8 ± 3.3 (3.0–3.0)	3.8 ± 3.4 (3.0–4.0)
Previous pregnancy n (%)	79 (46.5%)	74 (43.5%)
Total AFC	4.7 ± 2.0 (5.0–3.0)	4.4 ± 1.9 (5.0–3.0)
1–2	25 (14.7%)	32 (18.8%)
3–5	75 (44.1%)	85 (50.0%)
6–7	70 (41.2%)	53 (31.2%)
AMH (ng/ml)	0.94 ± 0.58 (0.81–0.63)	0.89 ± 0.55 (0.79–0.60)
Basal FSH values (mIU/ml)	7.66 ± 2.84 (7.03–2.91)	7.89 ± 3.21 (7.20–3.32)
Basal LH values (mIU/ml)	3.17 ± 1.74 (2.81–1.63)	3.35 ± 1.65 (2.91–1.77)
Basal E_2_ values (pg/ml)	38.21 ± 20.07	38.33 ± 16.07
	(32.5–19.0)	(35.0–21.0)
Basal *P*-values (ng/ml)	0.33 ± 0.22 (0.30–0.20)	0.34 ± 0.21 (0.30–0.20)
Indication of IVF *n* (%)		
DOR	16 (9.4%)	24 (14.1%)
DOR+ Tubal	87 (51.2%)	92 (54.1%)
DOR+ Male	17 (10.0%)	17 (10.0%)
DOR+ Endometriosis	24 (14.1%)	16 (9.4%)
DOR+ Others	26 (15.3%)	21 (12.4%)
Previous IVF attempts		
0	41 (24.1%)	55 (32.4%)
1–4	129 (75.9%)	115 (67.6%)

*Data are presented as mean ± SD (median-IQR) or n (%); IQR, interquartile range; BMI, body mass index; AFC, antral follicle count; AMH, anti-Mullerian hormone; DOR, diminished ovarian reserve*.

*No difference was found between the two groups (P > 0.05)*.

A total of 19 of 170 patients (11.2%) in the GnRH antagonist group and 17 (10.0%) in the PPOS group discontinued the trial or had a deviation from the protocol (*P* > 0.05) (Figure 1), so the per-protocol analysis did not include these cases. Four women in the GnRH antagonist group did not prescribe antagonist due to the short follicular phase, their follicles reached the mature criteria after 5–6 days' stimulation of gonadotropin, then arranged for oocyte retrieval. One case in PPOS group ovulated due to the accidentally delay of retrieval (at 38.9 h after trigger) and listed as protocol violation. A total of 24 cases (11 in antagonist group and 13 cases in PPOS group) did not continue to transfer their cryopreserved embryos. The data of full analysis set and per-protocol set had the similar results as ITT analysis (Tables S1, S2).

### Incidence of LH Rise and LH Surge

**Figure 2:**
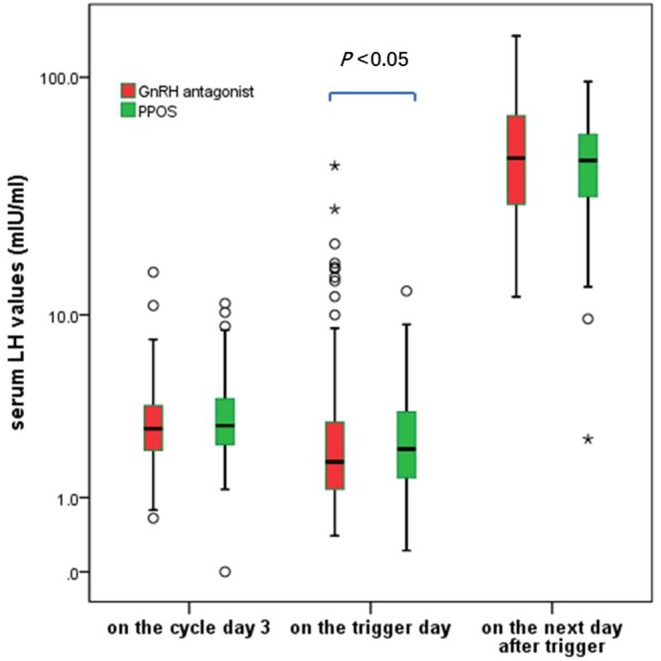
The serum LH fluctuation during the ovarian stimulation in the GnRH antagonist and PPOS groups. ^*^In the boxplot represents the extreme points.

GnRH antagonist group showed a wider spread of LH values on the trigger day (ranged from 0.40 to 43.18 mIU/ml in antagonist group and 0.51–12.74 mIU/ml in PPOS), the median of serum LH values on the trigger day in GnRH antagonist group was significantly lower than those in PPOS group (1.79 vs. 2.13 mIU/ml, *P* < 0.05) (Figure 2).

Twelve cases in GnRH antagonist group and one case in PPOS showed premature LH rise (LH > 10 mIU/ml) during the ovarian stimulation (7.1 vs. 0.6%, *P* < 0.05). Ten cases in the GnRH antagonist group experienced a premature LH surge (LH > 15 mIU/ml) but no case occurred in the PPOS group (5.88 vs. 0, *P* < 0.05), including three cases of premature LH surge occurred before the start of the GnRH antagonist treatment. Additionally, 2 cases in antagonist group unexpectedly ovulated at 36 h after trigger before oocyte retrieval although they did not show signs of premature LH rise. Another case presented elevated progesterone alone without LH rise or dominant follicle rupture in GnRH antagonist group and it was listed separately.

The average LH levels at 10–12 h after GnRH trigger were comparable between the two groups (*P* > 0.05). The cases with a suboptional pituitary response after trigger (post-trigger LH <15 mIU/ml) were distributed similarly between the two groups (4.1 vs. 2.9%, *P* > 0.05). These data showed the context of pituitary suppression using GnRH antagonist was more variable compared to that of PPOS in poor responders.

### Cycle Stimulation and Embryo Outcomes

The cycle characteristics and embryo results of the GnRH antagonist and PPOS cycles are shown in Table 2. The GnRH antagonist dose and duration were 0.70 ± 0.40 mg and 3.8 ± 1.9 days, respectively. The MPA duration was 8.9 ± 2.0 days. The dose and duration of hMG were comparable between the two groups (*P* > 0.05). The average E_2_ value on the trigger day was comparable between the two groups (1139.1 ± 740.2 vs. 1304.4 ± 903.9 pg/ml, *P* > 0.05).

**Table 2:** The IVF cycle and embryo characteristics between the two groups (ITT analysis).

	**GnRH antagonist** ** (*n* = 170)**	**PPOS** ** (*n* = 170)**	***P*-value**
hMG duration (days)	8.7 ± 2.7 (9.0–3.0)	8.3 ± 2.2 (8.0–3.0)	0.12
hMG dose (IU)	1,757.5 ± 677.2 (1,800.0–825.0)	1,636.2 ± 614.5 (1,800.0–825.0)	0.069
E_2_ values on trigger day (pg/ml)	1,139.1 ± 740.2 (936.0–933.0)	1,304.4 ± 903.9 (1,063.0–1,103)	0.33
FSH values on trigger day (mIU/ml)	14.09 ± 3.40 (13.81–5.08)	14.28 ± 4.0 (13.73–5.04)	0.75
LH values on trigger day (mIU/ml)	3.17 ± 4.84 (1.79–1.90)	2.59 ± 1.77 (2.15–2.05)	0.023
*P*-values on trigger day (ng/ml)	0.43 ± 0.30 (0.30–0.2)	0.40 ± 0.19 (0.40–0.2)	0.65
No. of>14 mm follicles on trigger day	3.4 ± 1.9 (3.0–3.0)	3.5 ± 2.2 (3.0–2.0)	0.88
Oocytes retrieved	3.4 ± 2.4 (3.0–3.0)	3.7 ± 2.6 (3.0–3.0)	0.30
MII oocytes	2.8 ± 2.2 (2.0, 3.0)	3.2 ± 2.4 (3.0–3.0)	0.12
Fertilized oocytes	2.2 ± 1.9 (2.0–2.0)	2.7 ± 2.2 (2.0–3.0)	0.063
Fertilization methods			0.08
ICSI	74 (44.3%)	53 (32.3%)	
IVF	86 (51.5%)	103 (62.8%)	
IVF+ICSI	7 (4.2%)	8 (4.9%)	
Cleavage embryos	2.2 ± 1.9 (2.0–2.0)	2.6 ± 2.1 (2.0–3.0)	0.057
Viable embryos	1.4 ± 1.3 (1.0–2.0)	1.6 ± 1.7 (1.0–2.0)	0.70
The proportion of viable embryos per oocyte	41.8% (244/584)	43.1% (273/633)	0.635

*Data are presented as mean ± SD (median-IQR) or n (%); IQR, interquartile range; hMG, human menopausal gonadotropin; ITT, intention to treatment*.

A total of 367 cases completed oocyte retrieval and 359 cases successfully harvested at least one oocyte. The numbers of retrieved oocytes were 3.7 ± 2.6 in PPOS group and 3.4 ± 2.4 in antagonist group, with the comparable proportion of immature oocytes, mature oocytes and abnormal oocytes (*P* >0.05). No between-group difference was found in terms of the number of fertilized oocytes or cleavage-stage embryos (*P* >0.05). The numbers of viable embryos used for transfer were comparable between the two groups (1.4 ± 1.3 vs. 1.6 ± 1.7, *P* > 0.05). The proportions of viable embryos per retrieved oocyte were similar between the two groups (41.8 vs. 43.1%, *P* > 0.05) (Table 2). In addition, no moderate/severe OHSS was recorded in either group.

Of 10 cases with LH surge in GnRH antagonist group, 3 cases with typical LH surge did not use trigger and harvested at least one mature oocyte on the next day, the other 7 cases were retrieved in advance based on the impending sign of ovulation, resulting into 6 cases with at least one mature oocyte. Three of the 10 cases produced at least one viable embryo and resulted into two live births.

Hundred and nineteen cases in the GnRH antagonist group obtained at least one viable embryo and 83 cases completed a fresh embryo transfer, resulting into 26 clinical pregnancies. Thirty-six cases were canceled fresh transfer due to the causes of the elevated progesterone values on the trigger day (*n* = 1), hydrosalpinx (*n* = 1), OHSS risk (*n* = 1), suboptimal endometrium (*n* = 15), or the patients' request (*n* = 18).

### Pregnancy Outcomes Between Two Protocols

Table 3 shows the pregnancy outcomes of embryo transfers using embryos originating from the GnRH antagonist and PPOS groups. A total of 238 transfer cycles had completed up to September 2018. The clinical pregnancy rate per women in the PPOS group was comparable to that in GnRH antagonist group (28.2 vs. 22.9%, RR 1.32 (95% CI 0.81, 2.16), *P* > 0.05). The miscarriage rate was comparable in all pregnancies of both groups (18.8 vs. 20.5%, RR 0.89 (95% CI 0.31, 2.59), *P* > 0.05). One case of ectopic pregnancy occurred in the PPOS group.

**Table 3:** Pregnancy outcomes after embryo transfer between the two groups (ITT analysis).

	**GnRH antagonist**			**PPOS** ** frozen ET**	*P*-value^*^
	**Fresh ET**	**Frozen ET**	**Total**		
Embryo transfer cycles	83	33	116	122	
Stages of embryo transferred					0.002
D2/3- no./total no. (%)	146 (100%)	42 (84.0%)	95.9% (188/196)	87.3% (178/204)	
D5/6- no./total no. (%)	0	8 (16.0%)	4.1% (8/196)	12.9% (26/204)	
No. of embryos transferred					
Mean	1.75 ± 0.44	1.53 ± 0.51	1.68 ± 0.49	1.67 ± 0.47	1.0
1—no./total no. (%)	24.1% (20/83)	51.5% (17/33)	31.9% (37/116)	32.8% (40/122)	
2—no./total no. (%)	75.9% (63/83)	48.5% (16/33)	68.1% (79/116)	67.2% (82/122)	
Clinical pregnancy per transfer (%)	31.3% (26/83)	39.4% (13/33)	33.6% (39/116)	39.3% (48/122)	0.36
Implantation rate (%)	19.9% (29/146)	26% (13/50)	21.4% (42/196)	29.4% (60/204)	0.067
Ectopic pregnancy rate (%)	0	0	0% (0/39)	2.1% (1/48)	1.0
Miscarriage rate (%)	15.4% (4/26)	30.8% (4/13)	20.5% (8/39)	18.8% (9/48)	0.84
Twin pregnancy rate (%)	11.5% (3/26)	0	8.8% (3/39)	29.2% (12/48)	0.034
Clinical pregnancy rate per woman (%)	26	13	22.9% (39/170)	28.2% (48/170)	0.26
Live birth rate per woman (%)	22	9	18.2% (31/170)	21.8% (37/170)^a^	0.42

* *Comparison between the two groups (116 cycles in GnRH antagonist group and 122 cycles in PPOS group)*.

a *One pregnancy woman in PPOS group was lost to follow up to delivery*.

*ITT, intention-to-treatment; ET, embryo transfer*.

The live birth rate was found no significant difference between the two groups (21.8 vs. 18.2%, RR 1.25 (95% CI 0.73, 2.13), *P* > 0.05). The birthweights of newborns were comparable between the two treatments and no congenital malformations were found in any of the liveborn babies.

**Figure 3:**
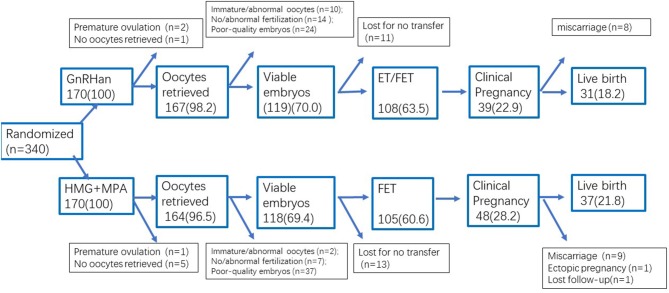
Transition probabilities for the overall trial.

The transition probabilities for the overall trial were shown in Figure 3.

## Discussion

In this open-label, single-central randomized controlled trial, we found that PPOS had a more robust effect at preventing premature LH surge than GnRH antagonist in poor responders (0 vs. 5.88%), and the two treatments showed similar efficacy of collecting competent oocytes (3.7 vs. 3.4), embryo yields (1.6 vs. 1.4), and probability of live birth in poor responders (21.8 vs. 18.2%).

Few studies introduced the mode of LH suppression or the pattern of circulating LH levels in poor responders using GnRH antagonist or PPOS cycles. The GnRH antagonist regimen allows higher LH levels in the early stimulation period, the LH levels rapidly decline after the start of the antagonist treatment in mid-follicular phase, often followed by a gradual increase later in the cycle (24, 25). In this trial, the LH values on the trigger day in GnRH antagonist group was lower than those in PPOS, with a bigger variance (0.40–43.18 mIU/ml), the premature LH surge occurred in 5.88% of all cases and another two cases ovulated unexpectedly before schedule. The transient LH suppression by GnRH antagonist was associated with competitive blockage of GnRH receptor, endogenous estrogen-induced GnRH release was still preserved, so that a small proportion of antagonist cycles failed to control the LH surge (26, 27). In contrast, progestin inhibits GnRH secretion on the hypothalamus if progestin is administered during the early part of the cycle before estrogen priming (28–30), the progestin administration shows an indirect, slow suppression of pituitary LH secretion, serum LH values are maintained at a relative steady level and oocyte retrieval could be easily programmed. Taken together with previous findings, PPOS showed an obvious superiority in controlling premature LH surge than GnRH antagonist in poor responders, but the differences in the pattern and the level of LH suppression might not play a role in the embryo yields and pregnancy outcomes.

The progestin's pituitary suppression during the controlling ovarian stimulation is still in the course of exploration. PPOS using different types of synthetic progestins (dydrogesterone, utrogestin and MPA) effectively suppressed the premature LH surge and produced a comparable number of viable embryos and pregnancy outcome (18, 19, 21, 31). In this trial, we chose MPA used for poor responders, for MPA did not interfere with the measurement of endogenous progesterone and seemed better than dydrogesterone for suppressing LH (21).

This trial differs from previous studies in several important ways. First, it is a well-designed RCT with the advantages of balancing multiple confounding factors in poor responders. Second, the participants still contained several follicles (total AFC 4.5 ± 1.9) and spontaneous menstrual cycles, they remained a certain extent ovarian function, which was a good population to investigate the efficacy of GnRH antagonist and PPOS in poor responders. Third, the primary objective is to compare the difference of progestin and GnRH antagonist used for preventing premature ovulation in poor responders, so the failure rate (premature LH surge) was listed as the primary outcome in this trial. The presence of LH surge during ovarian stimulation was considered as a warning signal for clinicians to make an optimal schedule timely although the optimal treatment was still a controversial problem (13, 14, 32). As for 10 cases with premature LH surge in antagonist group, the timing of oocyte retrieval was advanced according to the impending signs of ovulation and 9 cases captured at least one mature oocyte. The similar oocyte yields indicated the occurrence of premature LH surge did not comprise the results of GnRH antagonist group with the aid of flexible retrieval time. In addition, both FAS and PP analysis were completed in this trial and comparable results enhanced the conclusion.

The live birth is a good representation for the ultimate efficacy and significance of the two treatments. The two methods for controlling LH were subsequently accompanied by different embryo manipulations and transfer choices. GnRH antagonist protocol supported fresh embryo transfers if her conditions permitted, subsequently transferred surplus embryos. PPOS had to combine with freeze-all and delayed embryo transfer, so the results of this trial provided a real-world status about the two treatments for poor responders, rather than ideal protocols controlling all the confounding factors. The comparable pregnancy outcome indicated no significant difference for poor responders performing different types of pituitary suppression. The sample size of this trial was designed to investigate the difference of the incidence of LH surge, it had not enough power to distinguish the difference of live birth. Moreover, given that the limited space for increasing the probability of pregnancy for poor responders, a very large sample size is necessary to detect the difference of live birth in poor responders, so we should maintain precautions to generate the pregnancy outcomes.

These results are just a beginning of discussion for whether poor responders benefit most from PPOS or GnRH antagonist. Although PPOS is better in controlling LH levels than GnRH antagonist, PPOS in combination with delayed embryo transfer has to be in consideration of the disadvantages of increased economic cost by cryopreservation. The possible advantages of improved endometrial receptivity in FET should also be considered (33). Recent evaluation about cost-effectiveness demonstrated that PPOS may be well-suited for the high responders where freeze-only is likely and OHSS risk is high (34), and more evidences are needed to aid to choose the optimal treatment for poor responders. PPOS will be of significant public health value to pursue further research in this field. In addition, further work needs to identify specific values of PPOS for treating the refractory cases with premature ovulation during ovarian stimulation.

## Conclusion

PPOS had a more robust control for preventing premature LH surge than GnRH antagonist in poor responders. PPOS showed similar efficacy of collecting competent oocytes and embryos as GnRH antagonist in poor responders. PPOS in combination with freeze-all did not significantly increase the probability of pregnancy than GnRH antagonist protocol for poor responders. The two treatment strategies need further analysis using a large-sample well-designed trial to compare the live birth outcome and health economic significance.

## Data Availability Statement

All datasets generated for this study are included in the article/Supplementary Material.

## Ethics Statement

Human Ethics Committee approval for the research of PPOS and GnRH antagonist in poor responders came from the Institutional Review Board (IRB) of the Shanghai Ninth People's Hospital on 18 March 2017 (Number: 2016-198-T142). Chinese clinical trial registration number: ChiCTR-IPR-17010906, registered on March 18th 2017 (http://www.chictr.org.cn/showproj.aspx?~proj=11024).

## Author Contributions

QC supervised the entire study, including procedures, conception, design, and completion. LS and WC contributed to the data analysis and manuscript drafting. YW, RC, XZ, SZ, and XL were responsible for the collection of data. YK supervised the clinical trial. All authors participated in the ultimate interpretation of the study data and in revisions to the article.

### Conflict of Interest

The authors declare that the research was conducted in the absence of any commercial or financial relationships that could be construed as a potential conflict of interest.
