# Supplementary material for: Progestin vs. Gonadotropin-Releasing Hormone Antagonist for the Prevention of Premature Luteinizing Hormone Surges in Poor Responders Undergoing in vitro Fertilization Treatment: A Randomized Controlled Trial
Source: Front Endocrinol (Lausanne). 2019 Nov 22;10:796. doi: 10.3389/fendo.2019.00796 (PMC6882854; doi:10.3389/fendo.2019.00796)
Supplement: Supplementary file 1 [file Table_1.DOCX]

**Table S1 The cycle characteristics and pregnancy outcome in the two groups (FAS set)**

|  | GnRH antagonist  (n=166) | PPOS  (n=167) |
| --- | --- | --- |
| Age(years) | 35.1±4.1(36.0-6.0) | 34.8±4.2 (35.0-6.0) |
| BMI (kg/m^2^) | 21.7±2.7 (21.2-4.0) | 21.4±2.7 (21.1-4.0) |
| Duration of infertility(years) | 3.8±3.3 (3.0-4.0) | 3.8±3.5 (3.0-5.0) |
| Previous pregnancy n (%) | 78(47.0%) | 72(43.1%) |
| AMH (ng/ml) | 0.92±0.57(0.79-0.61) | 0.86±0.52(0.79-0.60) |
| Basal FSH value (mIU/ml) | 7.72±2.87 (7.07-2.85) | 7.93±3.23(7.24-3.39) |
| Total AFC | 4.6±1.9 (5.0-3.0) | 4.4±1.9 (4.0-3.0) |
| 0-2 | 25(15.1%) | 32(19.2%) |
| 3-5 | 75(45.2%) | 85(50.9%) |
| 6-7 | 66(39.8%) | 50(29.9%) |
| **Primary outcome** |  |  |
| Incidence of premature LH surge n (%) ^*^ | 10 (6.0%) | 0 |
| **Secondary outcome** |  |  |
| LH values on trigger day (mIU/ml) ^*^ | 3.24±4.91 (1.82-1.90) | 2.60±1.77(2.13-2.06) |
| E_2_ values on the trigger day (pg/ml) | 1127.62±728.49  (926.50-934.0) | 1268.38±853.64  (1061.0-1074.0) |
| No. of>14mm follicles on trigger day | 3.3±1.8 (3.0-3.0) | 3.4±2.0 (3.0-2.0) |
| Oocytes retrieved | 3.3±2.3 (3.0-3.0) | 3.6±2.4 (3.0-3.0) |
| Viable embryos | 1.4±1.3(1.0-2.0) | 1.6±1.6(1.0-2.0) |
| Embryo transfer cycles | 80 ETs/ 31 FETs | 120 FETs |
| Clinical pregnancy rate per transfer (%) | 32.4% (36/111) | 38.3% (46/120) |
| Implantation rate (%) | 20.9%% (39/187) | 27.5% (55/200) |
| Miscarriage rate (%) | 22.2% (8/36) | 19.6% (9/46) |
| Live birth rate (%) | 16.9% (28/166) | 21.0% (35/167) ^a^ |

Data are presented as Mean ± SD (Median-IQR) or n (%); IQR: interquartile range;

^a^ In PPOS group, one pregnancy was ectopic pregnancy and another one was lost to follow-up delivery outcome.

^*^ P<0.05; Other indexes were found no significant difference between the two groups (FAS set).
