# Supplementary material for: Progestin vs. Gonadotropin-Releasing Hormone Antagonist for the Prevention of Premature Luteinizing Hormone Surges in Poor Responders Undergoing in vitro Fertilization Treatment: A Randomized Controlled Trial
Source: Front Endocrinol (Lausanne). 2019 Nov 22;10:796. doi: 10.3389/fendo.2019.00796 (PMC6882854; doi:10.3389/fendo.2019.00796)
Supplement: Supplementary file 2 [file Table_2.DOCX]

**Table S2 The cycle characteristics and pregnancy outcome in the two groups (PP set)**

|  | GnRH antagonist  (n=151) | PPOS  (n=153) |
| --- | --- | --- |
| Age (years) | 34.9±4.2(35.0-6.0) | 34.7±4.2(35.0-6.0) |
| BMI (kg/m^2^) | 21.7±2.8(21.1-4.0) | 21.3±2.7 (20.7-4.0) |
| Duration of infertility(years) | 3.7±3.2 (3.0-3.0) | 3.9±3.5 (3.0-4.0) |
| Previous pregnancy n (%) | 73 (48.3%) | 64 (41.8%) |
| Antral follicle counts | 4.7±1.9 (5.0-3.0) | 4.4±1.9 (5.0-3.0) |
| 0-2 | 21(13.9%) | 29(19.0%) |
| 3-5 | 66(43.7%) | 78(51.0%) |
| 6-7 | 64(42.4%) | 46(30.0%) |
| AMH (ng/ml) | 0.94±0.58 (0.81-0.63) | 0.87±0.53 (0.79-0.61) |
| Basal FSH values (mIU/ml) | 7.69±2.80(7.02-2.60) | 7.93±3.29 (7.20-3.36) |
| **Primary outcome** |  |  |
| Incidence of premature LH surge n (%) ^*^ | 8(5.30%) | 0 |
| **Secondary outcome** |  |  |
| LH values on the trigger day (mIU/ml) ^*^ | 3.04±4.82 (1.74-1.60) | 2.49±1.54(2.11-2.09) |
| E_2_ values on the trigger day (pg/ml) | 1141.06±732.36  (940.0-938.0) | 1234.96±837.47  (1006.0-970.0) |
| No. of>14mm follicles on trigger day | 3.3±1.8 (3.0-3.0) | 3.4±2.0 (3.0-2.0) |
| No. of oocytes retrieved | 3.3±2.3 (3.0-3.0) | 3.6±2.5 (3.0-3.0) |
| Viable embryos | 1.4±1.3 (1.0-2.0) | 1.6±1.6(1.0-2.0) |
| Embryos transfer cycles | 80 ETs and 31 FETs | 120 FETs |
| Clinical pregnancy rate per transfer | 32.4% (36/111) | 38.3% (46/120) |
| Implantation rate (%) | 20.9% (39/187) | 28.5% (57/200) |
| Miscarriage rate (%) | 22.2% (8/36) | 19.6% (9/46) |
| Live birth rate (%) | 18.5% (28/151) | 22.9% (35/153) ^a^ |

Data was presented as Mean ± SD (median-IQR) or n (%). ^a^ In PPOS group, one pregnant case was ectopic pregnancy and one pregnant case was lost to follow up the delivery outcome.

^*^ *P*< 0.05; Other indexes were found no significance differences between the two groups (PP set).
